# Patient Experience of an Osteoporosis Telemedicine Clinic

**DOI:** 10.1093/geroni/igaa057.1396

**Published:** 2020-12-16

**Authors:** Melissa Steffen, Jennifer Van Tiem, Aaron Seaman, Karla Miller, Shylo Wardyn, Samantha Solimeo

**Affiliations:** 1 VA Office of Rural Health Veterans Rural Health Resource Center-Iowa City (VRHRC-IC), Iowa City, Iowa, United States; 2 Carver College of Medicine, University of Iowa, Iowa City, Iowa, United States; 3 VA Office of Rural Health Veterans Rural Health Resource Center-Salt Lake City (VRHRC-SLC), Salt Lake City, Utah, United States

## Abstract

Rural Veterans at risk of fracture due to osteoporosis remain underdiagnosed and undertreated, in part due to location-related barriers to accessing care. Despite lowered cost and travel barriers to osteoporosis care through implementation of a telehealth model directed at rural at-risk Veterans that took advantage of many strengths of the VA’s healthcare system, only 30% of eligible Veterans accepted care. To understand low acceptance, we conducted 39 semi-structured telephone interviews with Veterans eligible for the clinic, including 19 who accepted screening and treatment, 12 who completed screening but declined treatment, and 8 who declined screening and treatment. Veterans who opted to be screened and/or treated for osteoporosis did so because: it was recommended by the VA; they were interested in learning more about their health; thought they may be at risk of osteoporosis; or believed screening would not cause them harm. Conversely, Veterans refused screening or treatment because of past negative experiences with medications, both bone and non-bone; a wish to not put anything else into their bodies; or the belief that their bone loss is not severe enough to warrant treatment. Outside medical professionals and peers influenced Veterans’ decisions to not take or alter their treatment. Cost and travel distance remained a barrier for Veterans who did not live near a VA facility with the necessary screening and treatment infrastructure. Many barriers to osteoporosis care remain despite efforts to remove them. Delivery systems must account for both instrumental and social access to care to reduce fracture risk.

